# Supplementary material for: From sequence to enzyme mechanism using multi-label machine learning
Source: BMC Bioinformatics. 2014 May 19;15:150. doi: 10.1186/1471-2105-15-150 (PMC4229970; doi:10.1186/1471-2105-15-150)
Supplement: Additional file 2 — Java code of ml2db. Additional file ml2db_code.tar.gz contains the Java source code to run the multi-label machine learning experiments and save the results to database. The code’s Javadoc is included. [file 1471-2105-15-150-S2.zip › additional file 2/ml2db/ml2db/doc/main/ml2db/mulan/mulanarff/IdInstance.html]

IdInstance


JavaScript is disabled on your browser.


- Overview
- Package
- Class
- Use
- Tree
- Deprecated
- Index
- Help

- Prev Class
- Next Class

- Frames
- No Frames

- All Classes

- Summary:
- Nested |
- Field |
- Constr |
- Method

- Detail:
- Field |
- Constr |
- Method


main.ml2db.mulan.mulanarff

## Class IdInstance

- java.lang.Object
- - weka.core.AbstractInstance
  - - weka.core.SparseInstance
    - - main.ml2db.mulan.mulanarff.IdInstance

- All Implemented Interfaces:
  :   java.io.Serializable, java.lang.Comparable<IdInstance>, weka.core.Copyable, weka.core.Instance, weka.core.RevisionHandler

  Direct Known Subclasses:
  :   Neighbourhood

  ---

    

  ```
  public class IdInstance
  extends weka.core.SparseInstance
  implements java.lang.Comparable<IdInstance>
  ```

  A Weka instance with identifier.

  Version:
  :   11 Jul 2011

  Author:
  :   Luna De Ferrari ldf(at)st-andrews.ac.uk

  See Also:
  :   Serialized Form

- - ### Constructor Summary

    Constructors

    Constructor and Description

    `IdInstance(java.lang.String identifier,
    weka.core.Instance instance)`
  - ### Method Summary

    Methods

    | Modifier and Type | Method and Description |
    | --- | --- |
    | `int` | `compareTo(IdInstance otherInstance)` Order by instance identifier string |
    | `boolean` | `equals(IdInstance otherInstance)` |
    | `boolean` | `equals(weka.core.Instance otherInstance)` |
    | `java.lang.String` | `generateAllAttributeNamesString()` |
    | `java.util.Vector<java.lang.String>` | `generateNonZeroAttributesNames()` |
    | `java.lang.String` | `getString()` |
    | `weka.core.Instance` | `getWekaInstance()` |
    | `java.lang.String` | `identifier()` |
    | `void` | `setWekaInstance(weka.core.Instance wekaInstance)` |
    | `java.lang.String` | `toIdString()` Includes the attributes names. |
    | `java.lang.String` | `toSparseAttributesString()` |
    | `java.lang.String` | `toString()` |

    - ### Methods inherited from class weka.core.SparseInstance

      `copy, getRevision, index, locateIndex, main, mergeInstance, numAttributes, numValues, replaceMissingValues, setValue, setValueSparse, toDoubleArray, toStringNoWeight, value`
    - ### Methods inherited from class weka.core.AbstractInstance

      `attribute, attributeSparse, classAttribute, classIndex, classIsMissing, classValue, dataset, deleteAttributeAt, enumerateAttributes, equalHeaders, equalHeadersMsg, hasMissingValue, insertAttributeAt, isMissing, isMissing, isMissingSparse, numClasses, relationalValue, relationalValue, setClassMissing, setClassValue, setClassValue, setDataset, setMissing, setMissing, setValue, setValue, setValue, setWeight, stringValue, stringValue, toString, toString, value, valueSparse, weight`
    - ### Methods inherited from class java.lang.Object

      `equals, getClass, hashCode, notify, notifyAll, wait, wait, wait`

- - ### Constructor Detail


    - #### IdInstance

      ```
      public IdInstance(java.lang.String identifier,
                weka.core.Instance instance)
      ```
  - ### Method Detail


    - #### compareTo

      ```
      public int compareTo(IdInstance otherInstance)
      ```

      Order by instance identifier string

      **Specified by:**
      :   `compareTo` in interface `java.lang.Comparable<IdInstance>`


    - #### equals

      ```
      public boolean equals(IdInstance otherInstance)
      ```


    - #### equals

      ```
      public boolean equals(weka.core.Instance otherInstance)
      ```


    - #### generateAllAttributeNamesString

      ```
      public java.lang.String generateAllAttributeNamesString()
      ```


    - #### generateNonZeroAttributesNames

      ```
      public java.util.Vector<java.lang.String> generateNonZeroAttributesNames()
      ```


    - #### getString

      ```
      public java.lang.String getString()
      ```


    - #### getWekaInstance

      ```
      public weka.core.Instance getWekaInstance()
      ```


    - #### identifier

      ```
      public java.lang.String identifier()
      ```


    - #### setWekaInstance

      ```
      public void setWekaInstance(weka.core.Instance wekaInstance)
      ```


    - #### toIdString

      ```
      public java.lang.String toIdString()
      ```

      Includes the attributes names.

      Returns:
      :   public String toFullAttNamesString() { return this.identifier() +
          " " + this.toAttributeNamesString(); }


    - #### toSparseAttributesString

      ```
      public java.lang.String toSparseAttributesString()
      ```


    - #### toString

      ```
      public java.lang.String toString()
      ```

      **Overrides:**
      :   `toString` in class `weka.core.AbstractInstance`


- Overview
- Package
- Class
- Use
- Tree
- Deprecated
- Index
- Help

- Prev Class
- Next Class

- Frames
- No Frames

- All Classes

- Summary:
- Nested |
- Field |
- Constr |
- Method

- Detail:
- Field |
- Constr |
- Method
